# Supplementary material for: Suction-induced habitat selection in sand bubbler crabs
Source: R Soc Open Sci. 2019 May 22;6(5):190088. doi: 10.1098/rsos.190088 (PMC6549993; doi:10.1098/rsos.190088)

SUPPLEMENTARY MATERIALS

Suction-induced habitat selection in sand bubbler crabs

Shinji Sassa* and Soonbo Yang

INVENTORY OF SUPPLEMENTARY DATA

Supplementary Figure 1
Supplementary Figure 2
Supplementary Figure 3
Supplementary Figure 4
Supplementary Figure 5
Supplementary Figure 6

Supplementary Figure 1: Two-dimensional flume and experimental setup used for examining suction-induced habitat selection of sand bubbler crabs. Geoenvironmental gradients, namely suction gradients, were prescribed and varied by controlling the water levels in the reservoir on the left and right side of the flume, which changed the profiles of the groundwater levels in the sediment. Suction s denotes that at the level of the sediment surface. The five solid circles denote pore water pressure sensors, and the two open circles denote water pressure sensors.

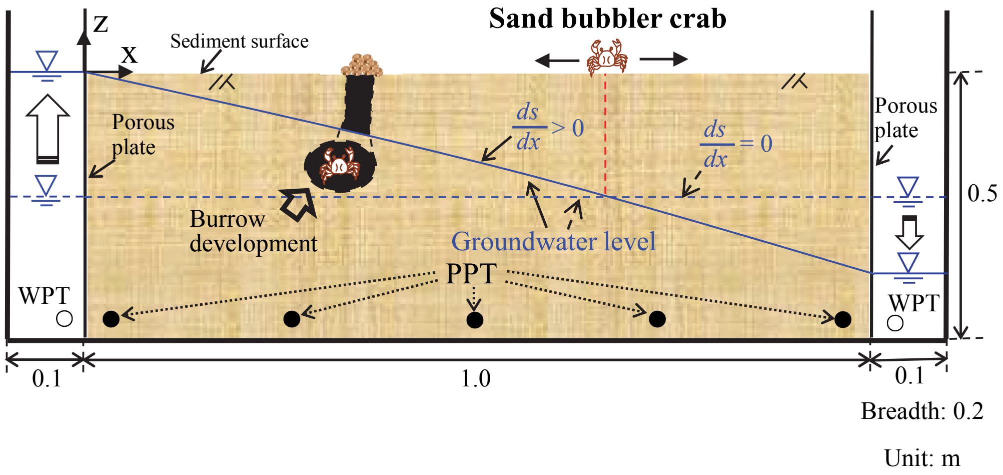

Supplementary Figure 2: Conceptual model to account for the link between burrow development, and suction and related geoenvironmental conditions [5]. OP^A and OP^B: optimum suction environments for the development of burrows above and below the groundwater level (G.W.L.), respectively. CR: critical suction environment for the development of burrows.

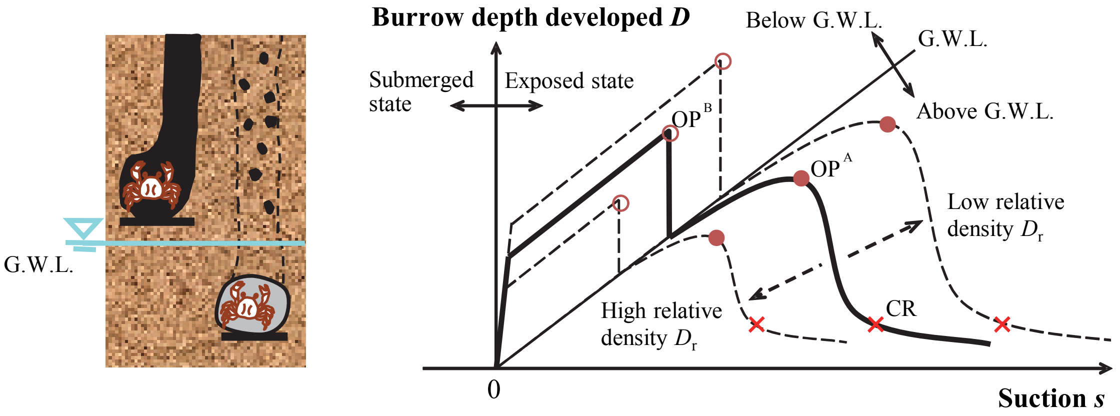

Supplementary Figure 3: Suction-induced habitat selection of sand bubbler crabs placed on the critical (CR) suction environment for the development of burrows. (*a*) Relationship between suction s and sediment hardness, which is vane shear strength τ^*. "Start Point" denotes the point where a sand bubbler crab was placed, and corresponding open symbols represent the points where crabs excavated burrows. Arrow shows the direction of movement. **(*b*)** No. of individuals excavating a burrow versus suction s. The majority of sand bubbler crabs placed on the CR suction environment moved toward the optimum (OP^B) suction environment, and the highest proportions of the individuals performed burrowing activities at the OP^B suction environment and in the vicinity.

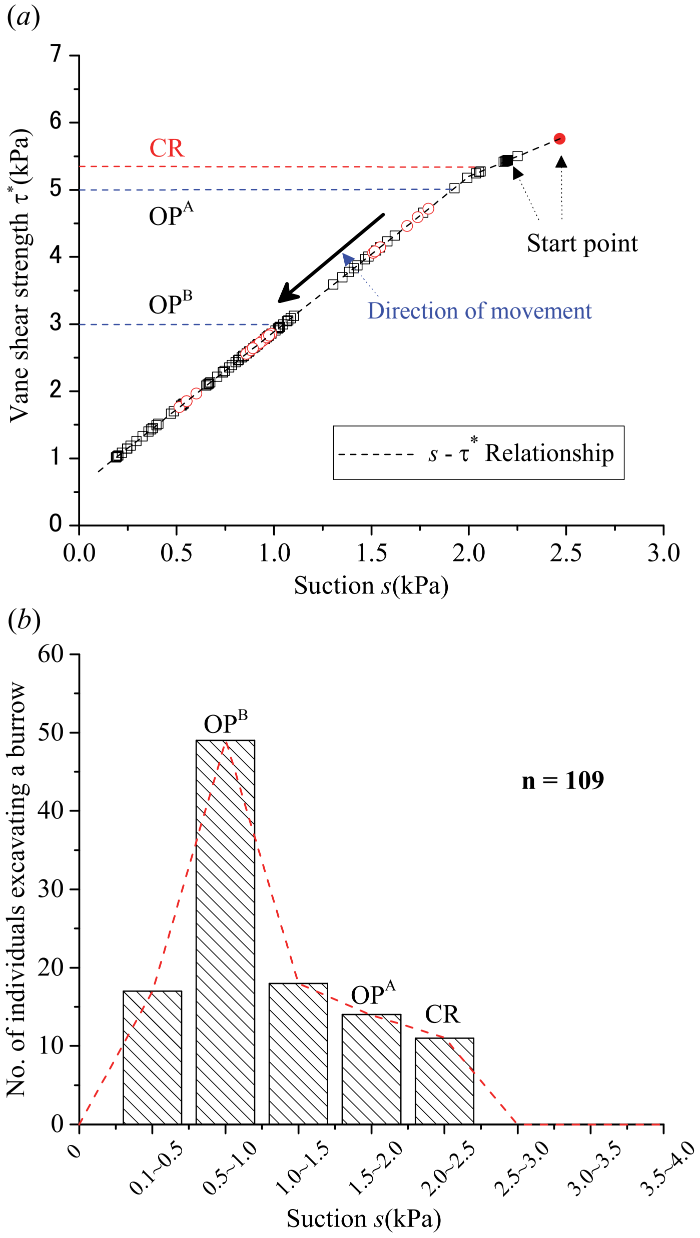

Supplementary Figure 4: Suction-induced habitat selection of sand bubbler crabs placed on the optimum (OP^A) suction environment for the development of burrows. (*a*) Relationship between suction s and sediment hardness, which is vane shear strength τ^*. "Start Point" denotes the point where sand bubbler crab was placed, and corresponding open symbols represent the points where crabs excavated burrows. Arrow shows the direction of movement. **(*b*)** No. of individuals excavating a burrow versus suction s. Sand bubbler crabs placed on the OP^A suction environment swiftly started burrowing there, or moved toward the more suitable optimum (OP^B) suction environment for the development of burrows. The highest proportions of the individuals performed burrowing activities at the OP^B suction environment and in the vicinity.

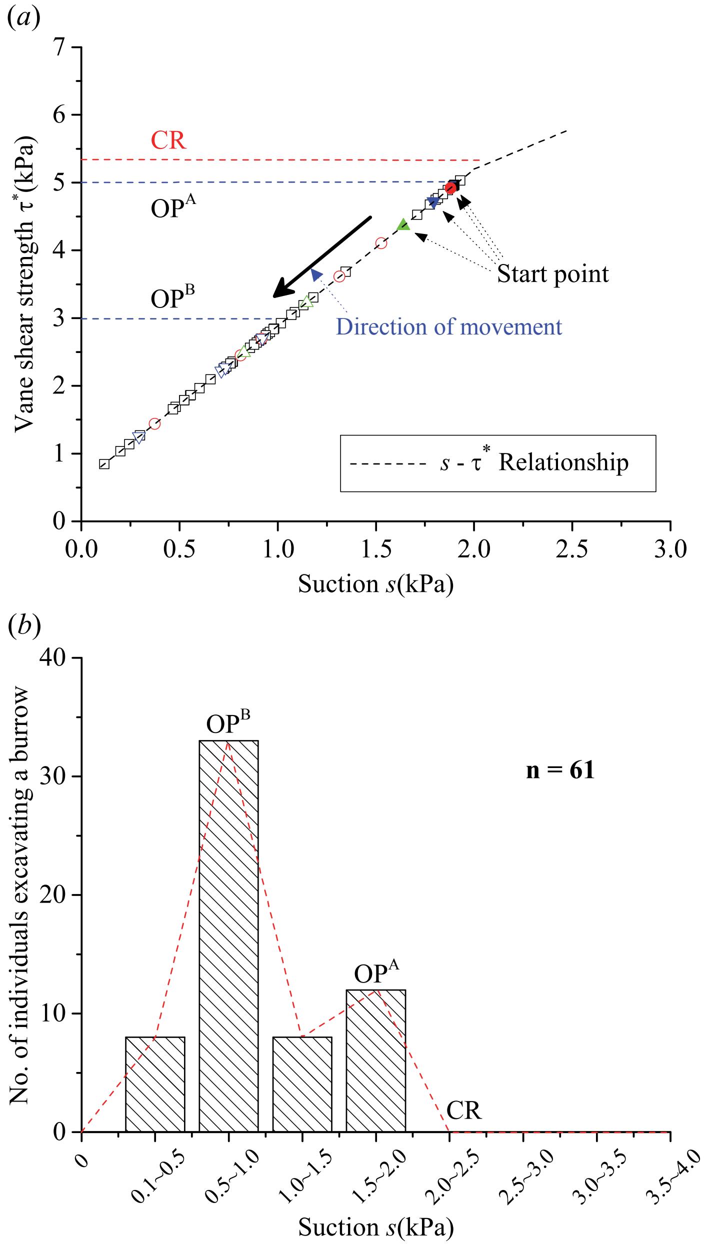

Supplementary Figure 5: Suction-induced habitat selection of sand-bubbler crabs placed on the optimum (OP^B) suction environment for the development of burrows. ***(a)*** Relationship between suction s and sediment hardness, which is vane shear strength τ^*. "Start Point" denotes the point where sand bubbler crab was placed, and corresponding open symbols represent the points where crabs excavated burrows. Arrow shows the direction of movement. ***(b)*** No. of individuals excavating a burrow versus suction s. All of the sand bubbler crabs placed on the OP^B suction environment immediately performed burrowing activities there and in the vicinity.

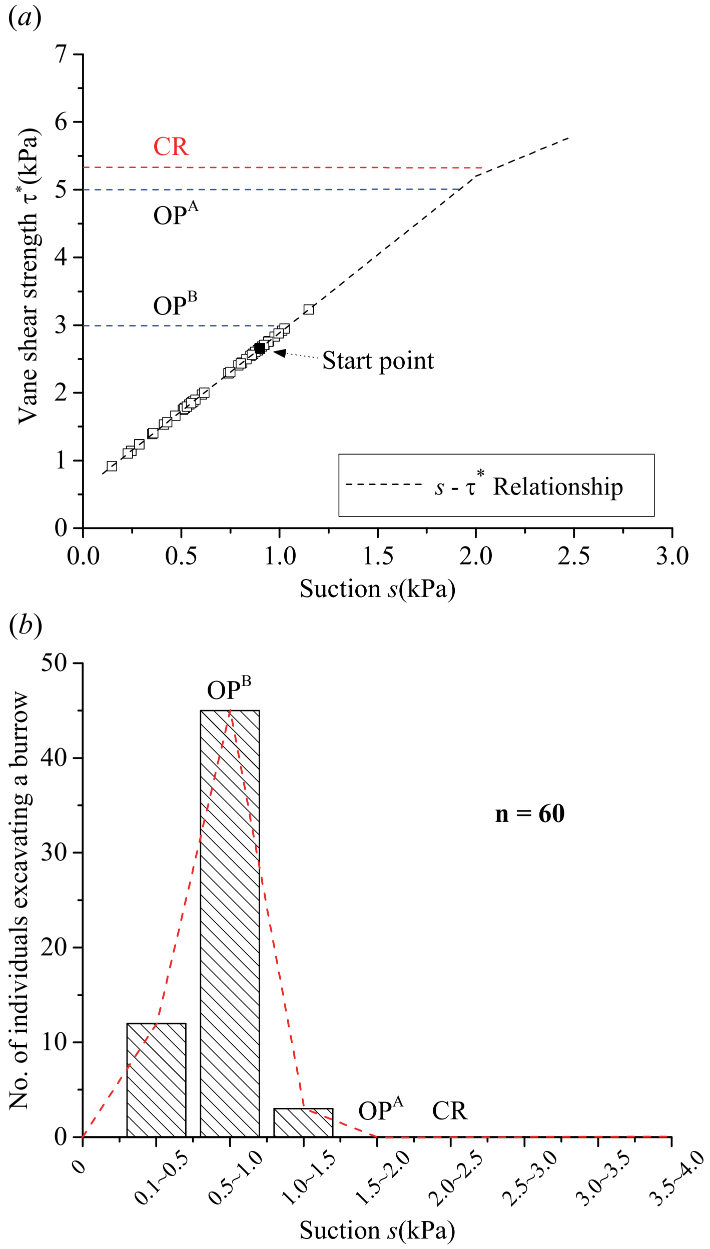

Supplementary Figure 6: Locations of the sandflats investigated in this study. The density surveys of sand bubbler crabs, instantaneous measurements of suction and groundwater level, and in situ undisturbed sediment samplings were conducted at Naha, Isumi and Nagura Amparu sandflats during spring low tides in the period from 2009 to 2015 with different seasons. At Tokuyama artificial sandflat, continuous measurements of suction and video recordings of sand-bubbler crab's behaviours were performed during periods of exposure on Sep. 28, 2015 and July 21, 2016, respectively. Laboratory experiments were conducted by using the intertidal sandy sediments taken from the Nojima sandflat.

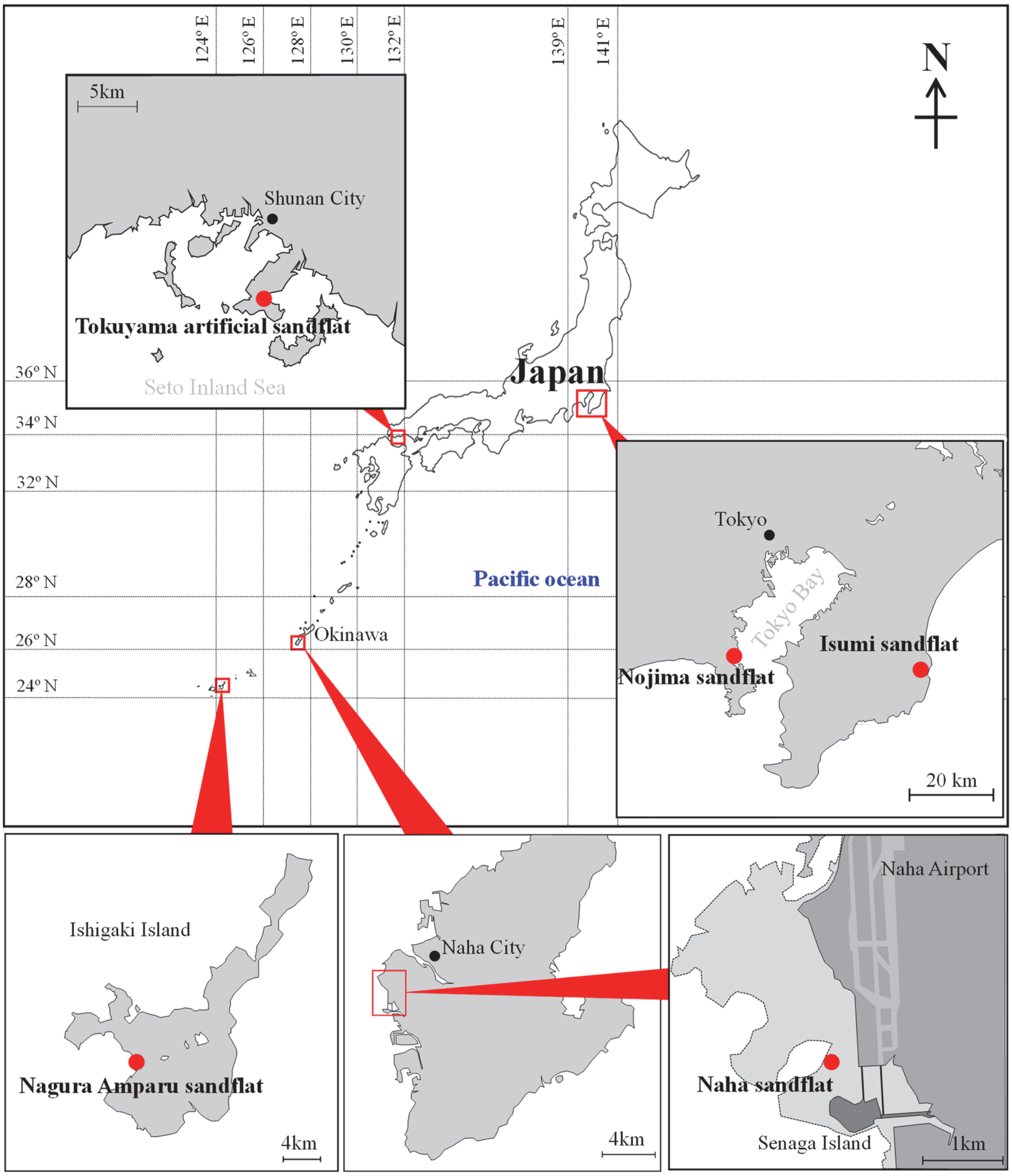

Supplement: Supplementary Figures 1-6 [file rsos190088supp1.pdf]
